# Association between Nutrient-Based Dietary Patterns and Bladder Cancer in Italy

**DOI:** 10.3390/nu12061584

**Published:** 2020-05-28

**Authors:** Valeria Edefonti, Carlo La Vecchia, Matteo Di Maso, Anna Crispo, Jerry Polesel, Massimo Libra, Maria Parpinel, Diego Serraino, Monica Ferraroni, Francesca Bravi

**Affiliations:** 1 Department of Clinical Sciences and Community Health, Università degli Studi di Milano, via A. Vanzetti, 5, 20133 Milan, Italy; valeria.edefonti@unimi.it (V.E.); carlo.lavecchia@unimi.it (C.L.V.); matteo.dimaso@unimi.it (M.D.M.); monica.ferraroni@unimi.it (M.F.); 2 Epidemiology and Biostatistics Unit, Istituto Nazionale Tumori—IRCCS “Fondazione G. Pascale”, via M. Semmola, 80131 Naples, Italy; a.crispo@istitutotumori.na.it; 3 Cancer Epidemiology Unit, Centro di Riferimento Oncologico di Aviano (CRO) IRCCS, via F. Gallini, 2, 33080 Aviano, Italy; polesel@cro.it (J.P.); serrainod@cro.it (D.S.); 4 Laboratory of Translational Oncology & Functional Genomics, Department of Biomedical and Biotechnological Sciences, Università di Catania, Via Santa Sofia, 97, 95123 Catania, Italy; m.libra@unict.it; 5 Department of Medicine, University of Udine, via Colugna, 50, 33100 Udine, Italy; maria.parpinel@uniud.it

**Keywords:** bladder cancer, case-control study, dietary patterns, diet, factor analysis

## Abstract

Limited knowledge is available on dietary patterns and bladder cancer risk. We analyzed data from an Italian case-control study carried out between 2003 and 2014, including 690 incident bladder cancer cases and 665 hospital-controls. We derived nutrient-based dietary patterns applying principal component factor analysis on 28 selected nutrients. We categorized factor scores according to quartiles, and estimated the odds ratios (ORs) and the corresponding 95% confidence intervals (CIs) through logistic regression models, adjusted for major confounding factors. We identified four dietary patterns named “Animal products”, “Vitamins and fiber”, “Starch-rich”, and “Animal unsaturated fatty acids”. We found an inverse association between the “Vitamins and fiber” pattern and bladder cancer (OR = 0.70, 95% CI: 0.48–0.99, IV versus I quartile category). Inverse relationships of borderline significance were also found for the “Animal products” and the “Animal unsaturated fatty acids” dietary patterns. No significant association was evident for the “Starch-rich” pattern. The current study allowed us to identify major dietary patterns in this Italian population. Our study confirms available evidence and shows that scoring high on a fruit-and-vegetables pattern provides beneficial effects on bladder cancer risk.

## 1. Introduction

Bladder cancer accounted for about 550,000 new cases worldwide in 2018 and ranked 15th among the causes of cancer mortality [1]. Tobacco smoking is the major recognized risk factor for this neoplasm, accounting for 28% of male cases and 18% of female cases in Europe, and 21% and 16%, respectively, in the USA [2]. Other well-known risk factors are past occupational exposures to aromatic amines, high level of arsenic in drinking water, and *Schistosoma haematobium* and other urinary tract infections, whereas the role of other lifestyle factors needs to be clarified [3,4,5]. Diet has been involved in the development of bladder cancer because the metabolites of the ingested foods have direct contact with bladder mucosa [6]. However, the role of specific foods and nutrients is still unclear, and the World Cancer Research Fund updated report indicates that only limited/suggestive evidence has been reached for a favorable role of vegetables and fruit consumption and tea drinking, while limited/non-conclusive evidence was available for the other dietary items [7].

Most studies have investigated single foods or nutrients, while only a few studies have used *a priori* [8,9,10,11] or *a posteriori* dietary patterns [12,13] to describe dietary habits as a complex behavior and to assess their relationship with bladder cancer risk. Previous analyses of selected aspects of diet in the present Italian population showed an unfavorable role of meat consumption (particularly stewed and roasted meat) [14], carbohydrates and glycaemic load [15], and an inverse relationship with vegetables, milk/yogurt [14], and flavones and isoflavones [16]. Considering dietary habits as a whole in the same population, bladder cancer was positively associated with a pro-inflammatory diet [17] and inversely related to a Mediterranean diet [18].

A national food consumption survey conducted in Italy in early 2000 showed that cereals represented the primary source of energy (providing ~38% of energy), followed by oils and fats (~17%, mainly from oils), and milk products (~13%); meats and fish provided a limited contribution (~9% and 2% of energy, respectively) [19]. Cereals were the primary source of fiber, providing a fiber intake comparable to the one given by the sum of fruit and vegetables [19]. However, the mean daily intake of vegetables, legumes, and fruit was below the recommendations provided by the World Cancer Research Fund [20].

To further investigate the role of overall dietary habits on bladder cancer risk, we derived *a posteriori* dietary patterns—using an exploratory principal component factor analysis—in an Italian case-control study.

## 2. Materials and Methods

### 2.1. Design and Participants

A hospital-based case-control study was carried out between 2003 and 2014 in four Italian areas —i.e., Milan, Pordenone, Naples, and Catania. The study design and inclusion criteria have been described in detail elsewhere [21]. Briefly, cases were 690 patients (including 595 men and 95 women) younger than 85 (age range: 25–84, median: 67) years with incident urothelial carcinoma of the bladder, recruited in major teaching and general hospitals of the study areas, and with no previous history of other neoplasms. Almost all cancer cases (*n* = 645, 93%) were confirmed by histology or cytology. Cases were classified on the basis of the 2016 World Health Organization (WHO) grading system [22]: 268 cases (38.8%) were non-muscle invasive (i.e., TNM pTis/pTa), 192 (27.8%) were pT1, 159 (23.0%) were muscle-invasive (i.e., pT2–pT4); as to the grading, 307 cases (44.5%) were moderately or well-differentiated (i.e., G1–G2), and 312 (45.2%) were undifferentiated or poorly differentiated (i.e., G3–G4). Controls were 665 patients (including 561 men and 104 women), selected among subjects admitted to the same hospital networks of cases for acute non-neoplastic diseases unrelated to alcohol drinking or tobacco smoking, and not associated with long-term dietary modifications. The median age of controls was 66, range 27–84 years. Among controls, 39.9% were admitted to hospital for acute surgery, 28.9% for traumas, 22.1% for nontraumatic orthopedic conditions, and 9.8% for other miscellaneous diseases. Less than 5% of cases and controls approached did not participate. The study protocol was submitted to the Board of Ethics of the participating hospitals and received the approval required at the time of data collection. The Ethics Committee of the National Cancer Institute “Centro di Riferimento Oncologico, IRCCS”, Aviano, updated the study approval on 14 December 2012 with the protocol number IRB-15-2012. The Ethics Committee of the Hospital “Niguarda Ca’ Granda”, Milan, provided the study approval on 23 March 2012, with register number 99_03/2012.

Details on selected characteristics of the study participants are provided in Table S1.

### 2.2. Data Collection

Participants’ data were collected during their hospital stay by centrally trained interviewers through a structured questionnaire assessing information on sociodemographic characteristics, anthropometric factors, tobacco smoking, a personal medical history of selected diseases, and occupational exposures. Dietary habits in the two years before cancer diagnosis (or before interview for controls) were also assessed with a food frequency questionnaire (FFQ), including 80 foods and recipes, and a list of different beverages. The FFQ included the following sections: (I) milk and sweeteners, (II) bread, cereals, and first courses, (III) second courses, (IV) side dishes (i.e., raw and cooked vegetables), (V) fruit, and (VI) sweets and desserts. Additional sections concerned lifetime consumption of beverages, including (a) alcoholic beverages, (b) hot beverages, (c) soft drinks, and (d) tap and bottled water [14,23]. Participants were asked to recall their usual frequency of consumption per week for each dietary item. Occasional consumption (defined as frequency >1 per month and <1 per week) was coded as 0.5 per week. For each participant, we estimated total energy, selected nutrient intakes, and the amount of condiments (e.g., butter and different types of oil) used in the recipes, through an Italian food composition database [24,25]. The validity and reproducibility of the FFQ were tested using a 7-day dietary record repeated twice [26,27].

### 2.3. Statistical Analysis

#### 2.3.1. Factorability of the Original Matrix

The analyses were carried out on a comprehensive list of 28 macronutrients, micronutrients, and minerals. We examined the potential relationships among nutrients to avoid over-representing any single nutrient or specific profiles of consumption, thus resulting in artificially higher correlation coefficients. We evaluated the factorability of the correlation matrix of the original nutrients (including both cases and controls) by visual examination of the matrix and through statistical procedures, namely Bartlett’s test of sphericity, overall (Kaiser–Meyer–Olkin) and individual measures of sampling adequacy [28]. Since we obtained satisfactory results (see Table 1), we used an exploratory principal component factor analysis to derive the *a posteriori* dietary patterns.

#### 2.3.2. Dietary Pattern Identification

We carried out a principal component factor analysis [29] on the correlation matrix of the 28 selected nutrients (including cases and controls together) to describe the variance–covariance structure of these variables in terms of a smaller number of underlying unobservable and randomly varying factors, which can be interpreted as dietary patterns. We selected the number of factors to retain, taking into account the following criteria: factor eigenvalue >1, scree-plot visual inspection, and factor interpretability [29]. We applied a varimax rotation to obtain a simpler and more interpretable loading structure. Each factor’s interpretation and labeling were based on nutrients with rotated factor loadings ≥0.63 in absolute value. We set this cut-off because it implies a minimum contribution of any factor to any nutrient’s total variance of approximately 40% (i.e., 0.63^2^) [30]. Nutrients with such factor loadings are called “dominant nutrients” hereafter. Factor scores, as continuous measures, were calculated for each participant and each pattern, and quantify the degree of adherence of each subject’s diet to the identified pattern. Factor scores were computed using the weighted least squares method [31].

#### 2.3.3. Reproducibility, Reliability, and Validity of Dietary Patterns

To evaluate the internal reproducibility of the identified dietary patterns, we performed additional analyses using a different procedure for estimating factor scores (namely, multiple regression method), and different estimation methods (namely, principal axis factor analysis with generalized least squares estimation method, and maximum likelihood factor analysis, after logarithmic transformation of the original nutrients) [29]. Moreover, following a split-half approach, we split the original dataset into 2 randomly selected subsamples (with cases and controls equally distributed), and carried out the principal component factor analysis procedure separately in each subsample; this procedure was repeated several times using different starting seeds for the random assignment, in order to verify the stability of the identified patterns [31]. As a sensitivity analysis, the principal component factor analysis procedure was also carried out among controls only.

Since all these checks were satisfactory, we performed all the subsequent analyses on factor scores obtained from the main analysis based on principal component factor analysis performed on cases and controls together, with varimax rotation and weighted least squares method.

To evaluate factor reliability and refine the identified dietary patterns, we calculated the standardized Cronbach’s coefficient alpha for those nutrients with a factor loading ≥0.40 in absolute value on any factor [32]. For each factor, we computed an overall coefficient alpha and several nutrient-specific coefficient alphas when-item-deleted, which assessed the importance of each nutrient within the corresponding pattern.

To further describe and interpret the identified dietary patterns, we calculated the Spearman rank correlation coefficients between the continuous factor scores derived from principal component factor analysis and the weekly intake of selected food groups and condiments, defined on the same data and derived from the original 80 food items [31].

#### 2.3.4. Risk Estimates

For each dietary pattern, we categorized participants into 4 groups according to the quartiles of the distribution of factor scores among cases and controls combined. We estimated the odds ratios (ORs) and the corresponding 95% confidence intervals (CIs) for quartile categories of factor scores compared to the reference category (i.e., the lowest quartile category for patterns characterized by positive factor loadings, and the highest quartile for patterns characterized by negative factors loadings), using unconditional multiple logistic regression models. We fitted both separate models for each factor and a composite model, including all the factors simultaneously. We included in each model the following potential confounding variables: age (<55, 55–59, 60–64, 65–69, 70–74, 75–79, ≥80 years), sex, center of recruitment, education (<7, 7–11, ≥12 years), cigarette smoking status and intensity (never, former, current <15, current 15–25, current ≥25 cigarettes/day), alcohol drinking intensity (<1, 1–<2, 2–<4, ≥4 drinks/day), history of occupational exposure (yes vs. no) in selected sectors relevant for bladder cancer risk, history of diabetes, history of cystitis, family history of bladder cancer, year of interview, and body mass index (BMI, <20, 20–24, 25–29, ≥30 kg/m^2^). The tests for linear trends were computed for all these models, scoring the quartile categories as numbers from 1 to 4. Moreover, we fitted a composite model including the factor scores of the identified dietary patterns in continuum (with measurement unit equal to 1 standard deviation), which estimates the mean variation in bladder cancer risk per 1 standard deviation increment of factor scores, accounting for the aforementioned confounding variables. 

Calculations were performed using the open-source statistical computing environment R (Ihaka and Gentleman, 1996; R Core Team, 2019), with its libraries psych [33] and GPArotation [34].

## 3. Results

Visual inspection of the correlation matrix of the original nutrient variables (among cases and controls) indicated that it was adequate to carry out a factor analysis. Table 1 shows the results from statistical procedures for checking matrix factorability. In particular, the Bartlett’s test of sphericity was statistically significant (*p*-value < 0.001), allowing to reject the null hypothesis that the correlation matrix is the identity matrix. The overall measure of sampling adequacy (Keiser–Meyer–Olkin statistic) was 0.86, thus indicating that the sample size was adequate compared to the number of nutrients included in the analysis. In addition, the individual measures of sampling adequacy were satisfactory for all considered variables, since 9 nutrients had measures ≥0.90, 12 had measures between 0.80 and 0.89, 5 had measures between 0.70 and 0.79, and only 2 nutrients had measures between 0.60 and 0.69.

Table 2 gives the factor loading matrix of the four selected dietary patterns and the corresponding communalities. The retained dietary patterns explained about 78% of the variance of the original nutrient variables. All the examined nutrients had one or more factor loadings ≥0.30, thus indicating that all the selected nutrients were relevant in this analysis. The greater (in absolute value) was the loading of a given nutrient to a factor, the higher was the contribution of that nutrient to the factor. The first dietary pattern was named “Animal products” and was characterized by high positive factor loadings on calcium, saturated fatty acids, riboflavin, animal protein, cholesterol, phosphorus, and zinc. The second one, labeled “Vitamins and fiber”, was characterized by high negative factor loadings on vitamin C, total fiber, beta-carotene equivalents, vitamin E, potassium, and total folate. The third dietary pattern, named “Starch-rich”, had high negative factor loadings on starch, vegetable protein, and sodium. The fourth one was labeled as “Animal unsaturated fatty acids” and showed high positive factor loadings on other polyunsaturated fatty acids and vitamin D.

The factor loading matrix derived from the sensitivity analysis on controls only is presented in Table S2. Visual inspection of the two-factor loading matrices provided reassuring results, with all the dominant nutrients highlighted in Table 2 being confirmed in Table S2. In addition, the single component and overall explained variances were comparable across the two solutions.

Standardized Cronbach’s coefficient alphas were high (at least 0.93) for all the factors, and most standardized coefficient alphas when-item-deleted were smaller than the corresponding coefficient alpha for the same factor, which supported the presence of internal consistency of the nutrients on each identified factor (data not shown).

Table 3 gives the Spearman correlation coefficients between the identified dietary patterns and selected food groups and condiments. The “Animal products” pattern score was positively correlated with the consumption of cheese, milk, liver, red meat, desserts, eggs, bread, and processed meat. The “Vitamins and fiber” pattern score had positive correlation coefficients with citrus fruit, other fruits, olive oil, fruiting vegetables, leafy vegetables, root vegetables, other vegetables, and pasta and rice. The “Starch-rich” pattern showed a week positive correlation with pasta and rice. The “Animal unsaturated fatty acids” pattern was positively correlated with the consumption of fish, unspecified seed oils, and red meat.

Table 4 shows the ORs and the corresponding 95% CIs for bladder cancer, according to quartile categories of the dietary patterns, and in continuum (per 1 standard deviation). Results refer to the composite models, including all the dietary patterns simultaneously. The “Vitamins and fiber” dietary pattern was inversely related to bladder cancer risk (OR = 0.70, 95% CI: 0.49–0.98, for the highest versus the lowest quartile category of consumption). Inverse relationships of borderline significance were also found for the “Animal products” dietary pattern (OR = 0.70, 95% CI: 0.48–1.01) and possibly for the “Animal unsaturated fatty acids” dietary pattern (OR = 0.81, 95% CI: 0.58–1.15). The remaining dietary pattern named “Starch-rich” was unrelated to bladder cancer risk (OR = 1.28, 95% CI: 0.90–1.81). Risk estimates in continuum were consistent with those in categories, indicating a mean risk reduction of 11% for the “Vitamins and fiber” pattern, and of 8% for the “Animal products” and “Animal unsaturated fatty acids” patterns. Risk estimates obtained from models fitted separately for each dietary pattern were comparable to those from the composite model (data not shown).

## 4. Discussion

In this case-control study on bladder cancer, we identified four major dietary patterns that explained almost 80% of the nutritional variability in this population. Among these, the “Vitamins and fiber” and possibly the “Animal products” and “Animal unsaturated fatty acids” patterns were associated with a decreased risk of bladder cancer, after mutual adjustment for all the remaining patterns. These patterns allowed to recover the main characteristics of the Italian diet at the time of the present study, in agreement with the findings of a national food consumption survey conducted in Italy in the same period [19].

Correlation coefficients between the identified patterns and selected food groups confirmed the labeling of dietary patterns and provided further insight into their composition. The “Animal products” pattern was primarily characterized by consumption of cheese and milk—which had the highest correlation coefficients—while minor, dominant components were different types of meat, eggs, and desserts (mainly bakery products and ice cream). The dairy products correlating with this pattern, especially milk, may have driven its inverse association with bladder cancer [14]. Moreover, in Italy, in contrast to Northern Europe and North America, poorer diets are largely based on bread, pasta, and carbohydrate-rich foods, instead of meat products, and meat consumption tends to be less unfavorable than elsewhere [35]. The “Vitamins and fiber” pattern correlated highly with different types of fruit and vegetables, as well as with olive oil; these dietary components are major sources of fiber, carotenoids, vitamin C, and vitamin E, and flavonoids, which have antioxidant and anti-inflammatory properties against cancer development [36]. Moreover, this is in line with findings supporting a favorable role of fruit and vegetables on bladder cancer, as indicated in the World Cancer Research Fund updated report [7]. The “Animal unsaturated fatty acids” pattern was mainly characterized by consumption of fish, and, to a lesser extent, by seed oils and red meat.

Evidence on dietary habits and bladder cancer risk is mainly based on single nutrients, foods, or food groups [7]. In the Danish Diet, Cancer and Health Study prospective cohort, no association was found between vitamin C, E, or folate and urothelial carcinoma, and a protective effect of dietary, but not supplemental, total beta-carotene was found [37]. Likewise, the Melbourne Collaborative Cohort Study found no association between dietary intake of B-group vitamins and urothelial cell carcinoma [38]. In the European Prospective Investigation into Cancer and Nutrition, no association was found between dietary folate, vitamin B2, B6, and B12 and urothelial cell carcinoma [39]. A population-based case-control study reported no association for fruits or vegetables, but an inverse association for vitamin B12 [40]. In our study, vitamin C, E, B-group, folate, and beta-carotene equivalents are all part of the same pattern named “Vitamins and fiber”, which was inversely related to bladder cancer risk.

The aforementioned case-control study also showed a positive association for processed meat intake [40]. A positive relationship between processed meat, and possibly red meat, and the risk of bladder cancer has also been reported in a meta-analysis, where, however, the association was evident in case-control but not prospective studies [41].

These findings confirm the difficulties of identifying a favorable or detrimental role of specific foods or nutrients on bladder cancer risk and the opportunity to consider diet as an overall exposure using the dietary pattern approach.

Only two papers from the same research group have reported on the role of food-based dietary patterns and bladder cancer risk [12,13]. In the first paper, the authors reported results of a case-control study, including 255 bladder cancer cases and 501 matched hospital controls from Uruguay [12]. They used factor analysis and identified 3 food-based dietary patterns, named “Sweet beverages”, “Western”, and “Prudent” patterns. The first one was characterized by high loadings on coffee, added sugar, and boiled eggs and was associated with an increased bladder cancer risk. In our study, we did not observe a pattern similar to that one. The “Western” pattern was characterized by positive loadings on red meat and wine and negative loadings on poultry and fish, and it was associated with an increased risk of bladder cancer. Given the common animal orientation, this pattern appears to have similarities with our “Animal products” and “Animal unsaturated fatty acids” patterns. However, unlike the Uruguayan pattern, ours were positively correlated with fish, and white and red meat; the presence of white meat and fish may explain the identified different results in terms of risk. The Uruguayan “Prudent” pattern was characterized by positive loadings on cooked and raw vegetables, citrus and other fruits, and desserts, and by negative loadings on French bread; this pattern was not related to bladder cancer risk. However, when the authors analyzed the separate effects on risk of the dominant food groups from the “Prudent” pattern, citrus fruit was inversely associated, whereas cooked vegetables were positively associated with bladder cancer; thus, the identified null association for that pattern appears to be the consequence of opposite effects on bladder cancer risk of its dominant food groups; an additional effect of condiments in cooked vegetables is likely to explain the positive association of this food group with bladder cancer risk. Another study was carried out by the same authors within a multi-site case-control design, including the aforementioned data on bladder cancer [13]. A factor analysis was carried out on two sets of male and female controls, and their association with cancer risk was evaluated separately by cancer site and sex. Four sex-specific and food-based dietary patterns called “Prudent”, “Traditional”, “Western”, and “Drinker” were identified. While the “Western” and “Prudent” patterns showed associations in the same direction observed in the previous study, the “Traditional” and “Drinker” patterns were unrelated to bladder cancer risk; however, CIs were, in general, very wide. To our knowledge, no studies have been published considering *a posteriori* dietary patterns derived on nutrients.

A few other studies have investigated the role of *a priori* dietary patterns on bladder cancer. In a previous analysis within our case-control study, we found an inverse association between adherence to the Mediterranean diet, as assessed through the Mediterranean Diet Score (MDS) and bladder cancer risk [18]. In a pooled analysis of 13 prospective studies from the BLadder cancer Epidemiology and Nutritional Determinants (BLEND) consortium, a higher MDS was associated with a reduction in bladder cancer risk [11]. In the Melbourne Collaborative Cohort Study (MCCS), the MDS and the Healthy Eating Index (HEI) showed borderline inverse associations with invasive, but not superficial, bladder cancers [9]; no relationship was found in the same study with the Dietary Inflammatory Index, representing a pro-inflammatory diet [9]. Similarly, in the Nurses’ Health Study (NHS) and in the Health Professionals Follow Up Study (HPFS), no association with bladder cancer was evident for the Empirical Dietary Inflammatory Pattern measuring the pro-inflammatory potential in diet [8]. A study within the Breast Cancer Detection Demonstration Project follow-up cohort reported an inverse association between the Recommended Food Score—a global measure of diet quality—and overall cancer risk, but no association was detected with bladder cancer [10].

Though these mixed results do not allow to draw firm conclusions, the role of diet in bladder carcinogenesis remains plausible; the biological argument targets those many compounds in foods and their metabolites that are excreted through the urinary tract [6].

Associations between dietary factors and disease may be influenced by selection and information bias, as well as confounding [42]. Hospital controls may not be fully representative of the general population. In our study, however, the low refusal rate and the comparable catchment areas of cases and controls avoided major selection biases. Furthermore, cases and controls were interviewed by the same trained personnel in the same hospital setting [43], and the FFQ was satisfactorily reproducible and valid [26,27], thus reducing the possibility of information bias. The FFQ aimed at assessing dietary habits two years before the enrolment in the study. While this time frame may be insufficient for the development of bladder cancer, it is implicitly assumed that most people do not appreciably change their dietary habits through their adult age; a bias may have occurred if this assumption did not hold. As for potential confounding, we were able to adjust for socioeconomic indicators, tobacco, and a number of other factors.

Further issues are related to the use of factor analysis to derive *a posteriori* dietary patterns [44,45,46]. This technique [47] requires subjective decisions at various levels of the analysis, including the type and number of dietary components to analyze, the number of factors to retain, the choice of applying a rotation method or not (and which method to use), and the interpretation of the identified factors [48]. For this reason, we performed several complementary analyses, which were reassuring and supported the (internal) reproducibility of the identified dietary patterns, as well as their interpretation.

## 5. Conclusions

The present work provides a comprehensive description of dietary habits in the Italian population considered, with the identification of four major dietary patterns explaining most of the variability in nutrient intakes. In line with the available evidence on dietary patterns and bladder cancer risk, the additional modeling effort required by dietary patterns has not ended up in stronger risk estimates for this cancer site, as compared to those observed for single dietary components. Our study confirms that scoring high on a fruit-and-vegetables pattern provides beneficial effects on bladder cancer risk.

## Figures and Tables

**Table 1:** Factorability of the correlation matrix of the original nutrients: Bartlett’s test of sphericity and measures of sampling adequacy.

**Bartlett’s Test of Sphericity:***p*-Value < 0.001	
**Overall Measure of Sampling Adequacy (Kaiser–Meyer–Olkin statistic) ^1^:** 0.86	
**Individual Measures of Sampling Adequacy:**	
0.60–0.69	retinol, linoleic acid
0.70–0.79	total fiber, starch, vitamin E, monounsaturated fatty acids, vitamin D
0.80–0.89	lycopene, vegetable protein, other polyunsaturated fatty acids, riboflavin, animal protein, saturated fatty acids, sodium, calcium, iron, vitamin C, potassium, folate
≥0.90	phosphorus, niacin, zinc, thiamin, cholesterol, soluble carbohydrates, linolenic acid, vitamin B6, beta-carotene equivalents

^1^ Overall and individual measures of sampling adequacy range between 0 and 1, with values > 0.60 indicating a satisfactory size.

**Table 2:** Factor loading matrix ^1^, communalities, and explained variances for the four major dietary patterns identified by principal component factor analysis.

Nutrient	Dietary Patterns				Communalities
	Animal Products	Vitamins and Fiber	Starch-Rich	Animal Unsaturated Fatty Acids	
Animal protein	**0.79**	−0.23	−0.20	0.43	0.90
Vegetable protein	0.13	−0.45	**−0.85**	0.18	0.97
Cholesterol	**0.78**	−0.12	−0.22	0.42	0.84
Saturated fatty acids	**0.82**	−0.26	−0.25	0.16	0.82
Monounsaturated fatty acids	0.48	−0.57	−0.24	0.33	0.72
Linoleic acid	0.46	−0.27	−0.25	0.45	0.54
Linolenic acid	0.60	−0.29	−0.23	0.31	0.59
Other polyunsaturated fatty acids	0.28	−0.19	−0.12	**0.87**	0.89
Soluble carbohydrates	0.43	−0.61	−0.16	−	0.59
Starch	0.13	−0.20	**−0.93**	0.14	0.94
Sodium	0.42	−	**−0.82**	−	0.87
Calcium	**0.83**	−0.30	−0.17	−	0.82
Potassium	0.48	**−0.72**	−0.30	0.25	0.90
Phosphorus	**0.77**	−0.39	−0.37	0.22	0.93
Iron	0.45	−0.49	−0.36	0.35	0.69
Zinc	**0.66**	−0.39	−0.43	0.42	0.94
Thiamin (vitamin B1)	0.56	−0.56	−0.41	0.25	0.85
Riboflavin (vitamin B2)	**0.82**	−0.40	−0.18	0.12	0.88
Vitamin B6	0.53	−0.61	0.29	0.39	0.89
Total folate	0.48	**−0.68**	−0.34	0.22	0.85
Niacin	0.40	−0.47	−0.33	0.58	0.83
Vitamin C	0.20	**−0.88**	−	−	0.82
Retinol	0.47	−	−	0.13	0.25
Beta-carotene equivalents	0.17	**−0.80**	−	0.23	0.73
Lycopene	−	−0.45	−0.22	0.37	0.40
Vitamin D	0.17	−0.18	**−**	**0.82**	0.74
Vitamin E	0.35	**−0.73**	−0.19	0.41	0.86
Total fiber (Englyst)	0.10	**−0.81**	−0.33	0.13	0.80
**Proportion of explained variance (%)**	26.59	24.31	13.88	13.31	
**Cumulative explained variance (%)**	26.59	50.90	64.78	78.09	

^1^ Estimates from a principal component factor analysis carried out on 28 nutrients, as measured among cases and controls together. For each factor, loadings greater or equal to 0.63 (in absolute value) indicated important or “dominant nutrients” in the current paper and were shown in bold typeface; loadings smaller than 0.1 in absolute value were suppressed.

**Table 3:** Spearman rank correlation coefficients ^1^ between continuous factor scores derived from principal component factor analysis on nutrient intakes and weekly number of portions for selected food groups and condiments derived on the same data.

Food Group	Animal Products	Vitamins and Fiber	Starch-Rich	Animal Unsaturated Fatty Acids
Milk	**0.45**	0.15	–	–0.18
Coffee	–	–	–	–
Tea and decaffeinated coffee	–	–	–	–
Bread	**0.35**	–	0.17	0.19
Pasta and rice	0.18	**0.32**	**0.29**	0.20
Soup	–	0.10	0.13	–
Eggs	**0.35**	–	0.13	0.18
White meat	0.18	0.13	0.10	0.14
Red meat	**0.38**	–	0.18	**0.41**
Liver	**0.40**	–0.03	–0.08	0.14
Processed meat	**0.32**	–0.02	0.19	0.13
Fish	–	–	–	**0.64**
Cheese	**0.63**	–	0.18	–
Potatoes	0.17	0.13	0.12	0.12
Pulses	–	0.24	0.16	–
Leafy vegetables	0.13	**0.40**	–	–
Fruiting vegetables	–	**0.45**	–	0.11
Root vegetables	0.06	**0.39**	–	0.10
Cruciferous vegetables	–	0.24	–	0.10
Other vegetables	0.23	**0.39**	–	0.10
Citrus fruit	–	**0.50**	–	–
Other fruits	–	**0.63**	–	–
Soft drinks and fruit juices	0.15	–	–	–
Desserts	**0.37**	–	0.17	–
Sugar and candies	0.24	0.22	–	–
Butter and margarine	0.24	–	–	–
Specified seed oils	–	–	–	0.18
Unspecified seed oils	0.17	–	–	**0.44**
Olive oil	0.12	**0.52**	0.11	0.11

^1^ Correlations greater or equal to 0.25 (in absolute value) were shown in bold typeface; correlations smaller than 0.1 (in absolute value) were suppressed.

**Table 4:** Odds ratios (ORs) ^1^ of bladder cancer and corresponding 95% confidence intervals (CIs) on quartiles of factor scores from a principal component factor analysis.

Dietary Pattern	Quartile Category, OR (95% CI)				*p* Trend ^3^	Per 1 SD ^4^
	I ^2^	II	III	IV		
**Animal products**	1	0.93 (0.66–1.30)	0.72 (0.50–1.03)	0.70 (0.48–1.01)	0.026	0.91 (0.80–1.04)
**Vitamins and fiber**	1	0.77 (0.55–1.09)	0.92 (0.66–1.30)	0.70 (0.49–0.98)	0.109	0.89 (0.79–1.01)
**Starch-rich**	1	1.25 (0.90–1.75)	1.50 (1.06–2.11)	1.28 (0.90–1.81)	0.107	1.02 (0.90–1.15)
**Animal unsaturated fatty acids**	1	0.82 (0.58–1.15)	0.58 (0.41–0.82)	0.81 (0.58–1.15)	0.084	0.91 (0.81–1.03)

^1^ Estimates from an unconditional logistic regression model adjusted for age, sex, center of recruitment, education, tobacco smoking, alcohol drinking, occupational exposure, history of diabetes, history of cystitis, family history of bladder cancer, year of interview, body mass index. Results refer to the composite model, including all the four factors simultaneously. ^2^ Reference category. ^3^ *p*-value for linear trend. ^4^ SD: standard deviation.

## Supplementary Materials

The following are available online at https://www.mdpi.com/2072-6643/12/6/1584/s1, Table S1: Distribution of 690 bladder cancer cases and 665 controls according to selected characteristics. Italy 2003–2014, Table S2: Factor loading matrix and explained variances for the four major dietary patterns identified by principal component factor analysis.
