# Supplementary material for: Association between Nutrient-Based Dietary Patterns and Bladder Cancer in Italy
Source: Nutrients. 2020 May 28;12(6):1584. doi: 10.3390/nu12061584 (PMC7353000; doi:10.3390/nu12061584)
Supplement: Supplementary file 1 [file nutrients-12-01584-s001.zip › Supplementary Table 1.docx]

**Supplementary Table 1.** Distribution of 690 bladder cancer cases and 665 controls according to selected characteristics. Italy 2003-2014

| **Characteristics** | **Cases N (%)** | **Controls N (%)** |
| --- | --- | --- |
| **Age** |  |  |
| <60 | 148 (21.5) | 178 (26.8) |
| 60-64 | 107 (15.5) | 119 (17.9) |
| 65-69 | 164 (23.8) | 147 (22.1) |
| 70-74 | 155 (22.5) | 124 (18.7) |
| ≥75 | 116 (16.8) | 97 (14.6) |
| **Sex** |  |  |
| men | 595 (86.2) | 561 (84.4) |
| women | 95 (13.8) | 104 (15.6) |
| **Center** |  |  |
| Aviano | 242 (35.1) | 250 (37.6) |
| Milan | 241 (34.9) | 238 (35.8) |
| Naples | 129 (18.7) | 100 (15.0) |
| Catania | 78 (11.3) | 77 (11.6) |
| **Education (years)^1^** |  |  |
| <7 | 292 (42.3) | 273 (41.1) |
| 7-11 | 224 (32.5) | 215 (32.3) |
| ≥12 | 173 (25.1) | 177 (26.6) |
| **Cigarette smoking^1^** |  |  |
| never | 96 (14.1) | 237 (35.6) |
| former | 310 (45.5) | 284 (42.7) |
| current, <15 cigarettes/day | 79 (11.6) | 53 (8.0) |
| current, 15-24 cigarettes/day | 127 (18.7) | 68 (10.2) |
| current, ≥25 cigarettes/day | 69 (10.1) | 23 (3.5) |
| **Alcohol drinking^1^** |  |  |
| <1 drink/day | 159 (23.1) | 184 (27.7) |
| 1-<2 drinks/day | 130 (18.9) | 113 (17.0) |
| 2-<4 drinks/day | 213 (30.9) | 222 (33.4) |
| ≥4 drinks day | 187 (27.1) | 145 (21.8) |
| **Body mass index (kg/m^2^)** |  |  |
| <20 | 19 (2.8) | 17 (2.6) |
| 20-24.9 | 230 (33.3) | 206 (31.0) |
| 25-29.9 | 316 (45.8) | 313 (47.1) |
| ≥30 | 125 (18.1) | 129 (19.4) |
| **History of cystitis** |  |  |
| no | 634 (91.9) | 630 (94.7) |
| yes | 56 (8.1) | 35 (5.3) |
| **History of diabetes** |  |  |
| no | 578 (83.8) | 608 (91.4) |
| yes | 112 (16.2) | 57 (8.6) |
| **Occupational exposure** |  |  |
| no | 401 (58.1) | 400 (60.1) |
| yes | 289 (41.9) | 265 (39.9) |
| **Family history of bladder cancer^2^** |  |  |
| no | 667 (96.7) | 654 (98.4) |
| yes | 23 (3.3) | 11 (1.6) |

^1^ The sum does not add up to the total because of the presence of missing values. ^2^ Family history of bladder cancer in first-degree relatives.
