# Supplementary material for: Association between Nutrient-Based Dietary Patterns and Bladder Cancer in Italy
Source: Nutrients. 2020 May 28;12(6):1584. doi: 10.3390/nu12061584 (PMC7353000; doi:10.3390/nu12061584)
Supplement: Supplementary file 1 [file nutrients-12-01584-s001.zip › Supplementary Table 2.docx]

**Supplementary Table 2.** Factor loading matrix and explained variances for the four major dietary patterns identified by principal component factor analysis.

| **Nutrient** | **Dietary patterns** | | | | **Communalities** |
| --- | --- | --- | --- | --- | --- |
|  | *Animal products* | *Vitamins and fiber* | *Animal unsaturated fatty acids* | *Starch-rich* |  |
| Animal protein | **0.77** | -0.22 | -0.51 | -0.25 | 0.96 |
| Vegetable protein | 0.16 | -0.46 | -0.28 | **-0.85** | 1.00 |
| Cholesterol | **0.76** | -0.13 | -0.44 | -0.27 | 0.86 |
| Saturated fatty acids | **0.81** | -0.28 | -0.27 | -0.33 | 0.92 |
| Monounsaturated fatty acids | 0.49 | -0.60 | -0.41 | -0.31 | 0.86 |
| Linoleic acid | 0.39 | -0.24 | -0.62 | -0.25 | 0.66 |
| Linolenic acid | 0.53 | -0.26 | -0.48 | -0.25 | 0.65 |
| Other polyunsaturated fatty acids | 0.25 | -0.16 | **-0.84** | -0.13 | 0.82 |
| Soluble carbohydrates | 0.43 | -0.61 | - | -0.25 | 0.62 |
| Starch | 0.14 | -0.23 | -0.20 | **-0.95** | 1.00 |
| Sodium | 0.41 | - | -0.16 | **-0.86** | 0.95 |
| Calcium | **0.83** | -0.31 | - | -0.26 | 0.85 |
| Potassium | 0.50 | **-0.72** | -0.34 | -0.31 | 0.98 |
| Phosphorus | **0.77** | -0.40 | -0.33 | -0.42 | 1.00 |
| Iron | 0.47 | -0.49 | -0.43 | -0.34 | 0.76 |
| Zinc | **0.66** | -0.40 | -0.51 | -0.47 | 1.00 |
| Thiamin (vitamin B1) | 0.55 | -0.57 | -0.29 | -0.44 | 0.90 |
| Riboflavin (vitamin B2) | **0.80** | -0.43 | -0.20 | -0.22 | 0.92 |
| Vitamin B6 | 0.54 | -0.60 | -0.44 | -0.30 | 0.94 |
| Total folate | 0.48 | **-0.71** | -0.30 | -0.35 | 0.95 |
| Niacin | 0.39 | -0.46 | -0.61 | -0.33 | 0.84 |
| Vitamin C | 0.24 | **-0.92** | -0.14 | - | 0.92 |
| Retinol | 0.49 | -0.11 | - | -0.10 | 0.27 |
| Beta-carotene equivalents | 0.17 | **-0.85** | -0.38 | - | 0.90 |
| Lycopene | 0.13 | -0.44 | -0.51 | -0.18 | 0.50 |
| Vitamin D | 0.12 | -0.15 | **-0.79** | - | 0.67 |
| Vitamin E | 0.34 | **-0.73** | -0.56 | -0.22 | 1.00 |
| Total fiber (Englyst) | 0.25 | **-0.81** | -0.19 | -0.35 | 0.83 |
| **Proportion of explained variance (%)** | 24.22 | 23.33 | 16.67 | 14.56 |  |
| **Cumulative explained variance (%)** | 24.22 | 47.55 | 64.22 | 78.78 |  |

^1^ Estimates from a principal component factor analysis carried out on 28 nutrients, as measured among controls only. For each factor, loadings greater or equal to 0.63 (in absolute value) indicated important or “dominant nutrients” in the current paper and were shown in bold typeface; loadings smaller than 0.1 in absolute value were suppressed.
